# Evolution of Enzyme Kinetic Mechanisms

**DOI:** 10.1007/s00239-015-9681-0

**Published:** 2015-05-19

**Authors:** Nuriye Nuray Ulusu

**Affiliations:** School of Medicine, Koç University, Rumelifeneri yolu, Sarıyer, Istanbul Turkey

**Keywords:** Enzyme kinetic mechanisms, Evolution, Substrate specificity, Structure, Function

## Abstract

This review paper discusses the reciprocal kinetic behaviours of enzymes and the evolution of structure–function dichotomy. Kinetic mechanisms have evolved in response to alterations in ecological and metabolic conditions. The kinetic mechanisms of single-substrate mono-substrate enzyme reactions are easier to understand and much simpler than those of bi–bi substrate enzyme reactions. The increasing complexities of kinetic mechanisms, as well as the increasing number of enzyme subunits, can be used to shed light on the evolution of kinetic mechanisms. Enzymes with heterogeneous kinetic mechanisms attempt to achieve specific products to subsist. In many organisms, kinetic mechanisms have evolved to aid survival in response to changing environmental factors. Enzyme promiscuity is defined as adaptation to changing environmental conditions, such as the introduction of a toxin or a new carbon source. Enzyme promiscuity is defined as adaptation to changing environmental conditions, such as the introduction of a toxin or a new carbon source. Enzymes with broad substrate specificity and promiscuous properties are believed to be more evolved than single-substrate enzymes. This group of enzymes can adapt to changing environmental substrate conditions and adjust catalysing mechanisms according to the substrate’s properties, and their kinetic mechanisms have evolved in response to substrate variability.

## Introduction

Life depends on a never-ending series of biochemical reactions, which are accomplished by enzymes (Martin 2011; Ulusu 2015). Enzymes are the catalysts of biological systems. They are extremely well organised and efficient. A typical enzyme accelerates the rate of a reaction by factors of at least a million compared to the rate of the same reaction in the absence of the enzyme. Almost every biochemical reaction depends on enzymatic reactions in cells (Martin 2011; Ulusu 2015; Wienkers and Rock 2014). These reactions culminate in the transformation of various chemicals according to metabolic needs, the transformation of chemicals into cellular useable forms, the detoxification of chemicals, the storage of chemicals as energy or the use of chemicals as signalling molecules for controlling metabolic pathways. Enzymes in living systems are continuously exposed to novel substrates from the sub-environment, and this flow of substrates affects the metabolic rate (Martin 2011; Ulusu 2015; Wienkers and Rock 2014; Miles et al. 2014). Catalysed molecules may be natural substrates, or they may be foreign molecules, such as toxins, drugs or insecticides (Martin 2011; Ulusu 2015; Wienkers and Rock 2014; Miles et al. 2014; Tevatia et al. 2014). The amino acid sequences of proteins exhibit diversity during evolution, as their structure dictates their function, which is crucial in sustaining life (Grishin 2001). The laws of physics and chemistry determine the properties of all molecules (Harms and Thornton 2013). In addition, random mutagenesis can create novel enzymes, proteins, entire metabolic pathways and even whole genomes with desired or improved capabilities (Labrou 2010).

Enzymatic reactions can result in the synthesis of novel molecules from novel ligands and assist in developmental processes in evolution and the continuation of life (Miles et al. 2014).

## Complexity of Kinetic Mechanisms

Kinetic models are among the tools that can be used for optimisation of bio-catalytic reactions, as well as for facilitating process design and upscaling to improve productivity and reduce the cost of various processes (Bornadel et al. 2013). Kinetic studies in enzymology can be classified into three categories: transient-state kinetics, steady-state kinetics and rapid-equilibrium kinetics (Segel 1975). Transient-state kinetics deals with very rapid reactions. The reaction mechanisms are directly related to the structure of the enzyme (Alberty 2010a; Fisher 2013). Steady-state enzyme kinetics are based on the assumption that the steps in the catalytic mechanism follow steady-state kinetics, with all the state variables remaining constant, despite exposure to continuous changes (Martin 2011). In rapid-equilibrium kinetics, prior to the rate-determining reaction, the reactions are in equilibrium with their components, such as the enzyme, substrate and enzyme-substrate complex (Alberty 2010b). According to Alberty, modifiers of enzyme-catalysed reactions have numerous effects on the velocity of a reaction (Alberty 2010a, b). When a single molecule of a modifier is bound to an enzyme, the kinetic mechanism determinants change and yield two rate constants. On the other hand, when two molecules of modifiers participate in two reactions, there are five independent equilibria and three paths for synthesising products (Alberty 2010b). Under laboratory in vitro conditions, drugs, toxins, radicals, activators, heavy metals and pH exert a major effect on the attainment of chemical equilibrium. However, the kinetic actions of enzymes are quite different under cellular conditions because of numerous variables (Cornish-Bowden 1999).

Various molecules have the ability to affect the kinetic mechanisms and behaviour of enzymes. Kinetic measurements can be used to predict the optimum kinetic behaviour, in other words, the best kinetic mechanism of a particular enzyme. Based on those predictions, the regulation of the enzyme by its substrates and products can be demonstrated. Studies have described the kinetic mechanisms of various enzymes, such as glutathione reductase (GR) and glucose-6-phosphate dehydrogenase (G6PD) purified from numerous tissues. These studies used double-reciprocal plots of the substrate and product inhibition assays to explain the kinetic behaviour of enzymes. They presented equations describing the rate of the reaction in terms of substrate and product levels and rate constants. Product inhibition studies are important to determine the type of enzyme kinetic mechanism. In a study of G6PD enzyme catalysis in sheep kidney cortex, research showed that the conversion of its substrate glucose-6-phosphate to its product occurred via a ping-pong mechanism, in which the product was released following the entry of two subsequent substrates into the reaction. On the other hand, the same enzyme isolated from lamb kidney cortex followed ordered bi–bi sequential kinetics, involving the binding of glucose-6-phostphate (G6P) to the free enzyme, followed by NADP^+^ binding. In bovine lens cortex, G6PD also adopted ordered bi–bi sequential kinetics. In contrast, in sheep brain cortex, it followed a Theorell–Chance mechanism. The distinct kinetic mechanisms highlight the various enzyme modifications that have taken place, including post-translational modifications at the molecular level (Ulusu et al. 1999, 2005; Ulusu and Tandogan 2006, 2007; Tandogan and Ulusu 2010; Ulusu and Sengezer 2012).

Studies of protein structure provide information underlying the principles of protein design that have come into play in natural evolution (Fleishman and Baker 2012). This information can be exploited in the redesign of enzymes for novel functions. The structure of the glutathione-binding domain of glutathione transferases is similar to that of other glutathione-linked proteins, such as glutathione peroxidases and thioredoxin, suggesting divergent evolution from a common ancestral protein fold (Fleishman and Baker 2012; Mannervik et al. 1998). Glutathione-dependent catalysis is a metabolic adaptation to chemical challenges encountered by all life forms. In the course of evolution, nature has optimised numerous mechanisms to use glutathione as the most versatile nucleophile for the conversion of a plethora of sulphur-, oxygen- and carbon-containing electrophilic substances (Mannervik et al. 1998; Deponte 2013). Glutathione-dependent enzymes are excellent for studying structure–function relationships and molecular evolution (Deponte 2013). The kinetic behaviour of GR isolated from various sources, such as cyanobacterium *Anabaena* sp. strain 7119 (Serrano et al. 1984), *Escherichia coli* (Bashir et al. 1995) and rat liver, exhibits a steady-state kinetic pattern typical of a ping-pong reaction mechanism. However, the aforementioned studies did not include any analyses of product inhibition kinetics. Such studies could shed light on the kinetic mechanism of GR enzymes (Carlberg and Mannervik 1975). Model simulations are consistent with the experimental observation that GR operates via both ping-pong and sequential branching mechanisms based on the concentration of its reaction substrate oxidised glutathione (GSSG) (Pannala et al. 2013). GR may change from using a sequential mechanism to a ping-pong mechanism (Rakauskiene 1989) or a hybrid ping-pong semi-random mechanism (Ozer and Ogus 2001). Ping-pong or sequential mechanisms of GR or those of other enzymes that act similar to GR are referred to as branched kinetic mechanism (Mannervik 1973).

The evolution of the kinetic mechanisms of enzymes included two important steps. The first was the catalytic promiscuity of substrates (Pandya et al. 2014). This property of enzymes is a widespread, but poorly understood, phenomenon among enzymes and is particularly relevant to the evolution of new functions, such as drug metabolism (Abhinav and Atkins 2008). Natural selection generally produces specific and efficient enzymes with broadened substrate specificity or enhanced catalytic promiscuity (O’Loughlin et al. 2006). Therefore, numerous enzymes can metabolise structurally distinct substrates or convert a single substrate to multiple different products. The ability to utilise one substrate to obtain several products for different cellular purposes is very important, and it is increasingly appreciated that functional promiscuity is important for the evolution of new protein functions (Ulusu 2015; Abhinav and Atkins 2008; O’Loughlin et al. 2006). The second important step in the evolution of the kinetic mechanisms of enzymes was the ability of enzymes to utilise novel ligands as natural substrates. This key property is very important for the survival of organisms. Glutathione S-transferases (GST) use the synthetic substrate 1-chloro-2,4-dinitrobenzene very efficiently (Loscalzo and Freedman 1986). However, these detoxification enzymes also have relatively high glutathione-conjugating activity for 4-hydroxynonenal, an electrophilic aldehyde derived from lipid peroxidation (Singh et al. 2001). Enzymes adopt a specific dimensional structure consisting of multi-enzymatic complexes (one-, two- or three-enzyme bio-catalysis), enabling kinematic reactions to be catalysed in a very short time. For example, the mammalian fatty acid complex has multiple domains, which function via distinct but linked enzymes (Chirala and Wakil 2004). The tight regulation of lipid levels can be accomplished, which is critical for cellular and organismal homoeostasis, not only in terms of energy utilisation and storage, but also to prevent potential toxicity (Karagianni and Talianidis 2015). The use of novel ligands as substrates in enzymatic catalysis yields novel products that can be used by organisms in innovative ways.

## Need for Different Kinetic Mechanisms

**Fig. 1:**
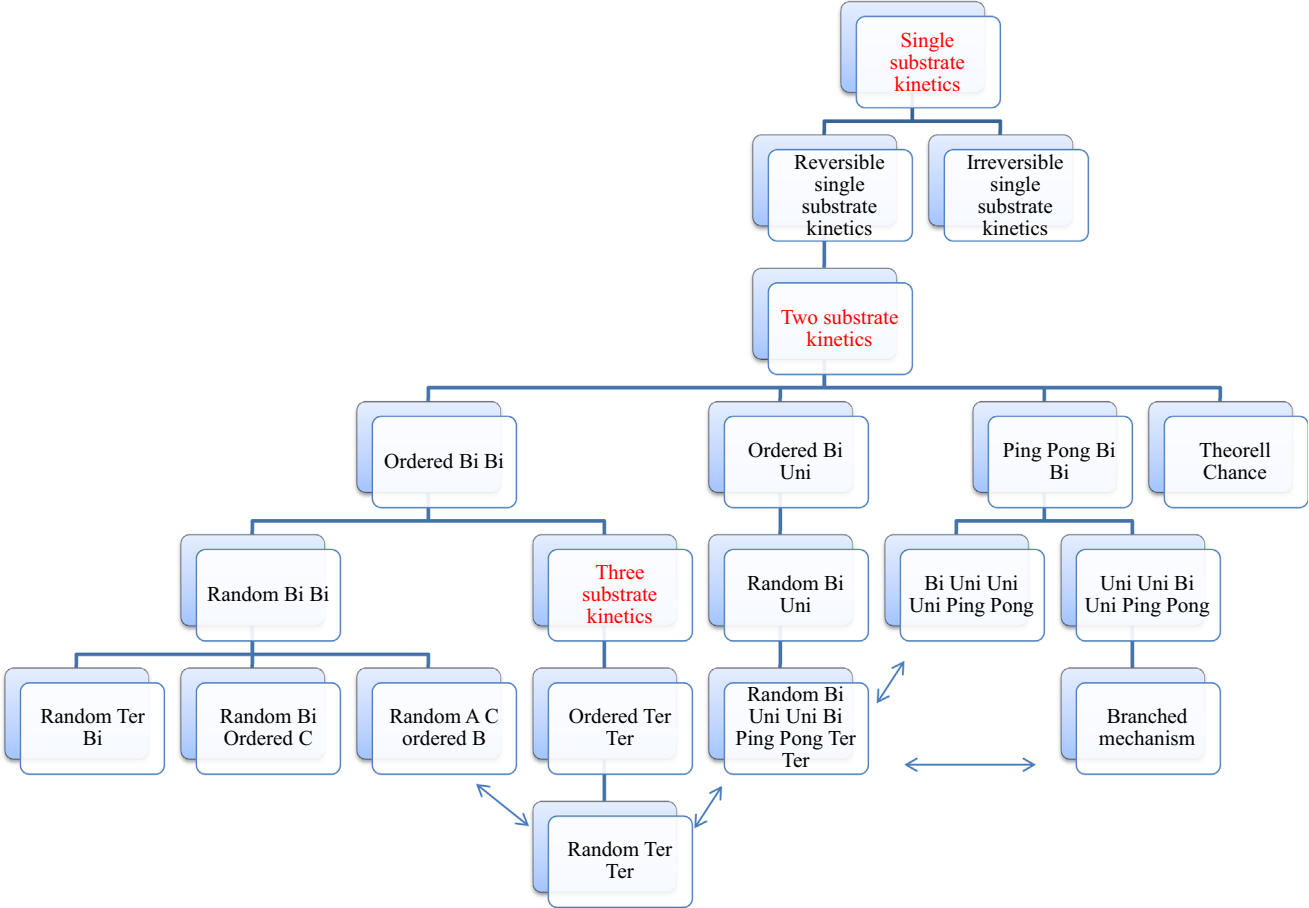
Possible evolutionary order of kinetic mechanisms. The figure schematically shows the going from *top* to *bottom* represents an evolutionary advance of kinetic mechanisms both of complexity and time

Enzymatic reactions proceed through a series of steps. These steps can shed light on the enzyme’s properties. Some enzymes have single-substrate molecules, such as hammerhead ribozymes (Murray et al. 2002) or proteases (Vitte 2015) According to the RNA world hypothesis, the early evolution of life depended on some RNA sequences catalysing the type of polymerisation needed for RNA replication (Benner 1989). Simple kinetic mechanisms are thought to have evolved first in ribozymes or protease enzymes (Murray et al. 2002; Vitte 2015). Single-substrate kinetic mechanisms are thought to represent the first steps in evolutionary processes (Johnston et al. 2001). In reality, most enzymes have complex active centres and have more than one substrate and more than one product. The complex biological activity of enzymes requires extraordinarily complex machinery, and the activity proceeds via very complex reactions. Enzymes that can catalyse complex reactions have multiple substrates and complex enzyme kinetic mechanisms. For enzymes with two substrates, the binding of these substrates can occur through two mechanisms: a sequential mechanism and a non-sequential mechanism. If the substrate forms an enzyme–substrate complex before a reaction takes place, the products that are released are called ‘sequential’. Sequential mechanisms have displacement reaction; both substrates bind to the enzyme and then reaction begins and proceeds to form products which are then released from the enzyme. Sequential mechanisms consist of three subgroups: random, ordered and Theorell–Chance types. In random mechanisms, any substrate can bind first to the enzyme, and any product can be produced. Theorell–Chance mechanism in which there is an obligatory order of substrate association and product release without the accumulation of the ternary complex. In ordered mechanisms, substrates are added and products are produced in a specific order. Non-sequential mechanism is also known as the “ping-pong” mechanism is characterised by the change of the enzyme into an intermediate form. The reaction proceeds with the release of one or more products between the additions of two substrates. This mechanism is also called the double placement reaction and common in group transfer. One key character of this reaction is the existence of a substituted enzyme intermediate, in which the enzyme is temporarily modified. The possible evolutionary order of these kinetic mechanisms is given in Fig. 1 (Murray et al. 2002; Vitte 2015; Benner 1989; Wang and Wu 2007; Zuccotti et al. 2001; McClard et al. 2006; Yu et al. 2014; Freist and Sternbach 1984; Celeste et al. 2012; Kim and Kang 1994; Menefee and Zeczycki 2014; Vergnolle et al. 2013).

Two or more enzymes (or multiple forms of the same enzyme) catalyse the same reaction. The substrate concentration determines the velocity of the enzyme reaction (Nagao et al. 2014; Wolfe 2005). In random-reaction mechanisms, the order in which the substrates bind does not matter. In ordered reactions, one substrate must bind the enzyme before the second substrate is able to bind (Segel 1975). The Theorell–Chance catalytic mechanism, also known as ‘hit-and-run’, is a specific type of ordered mechanism. The main difference between the Theorell–Chance mechanism and the ordered bi–bi mechanism is that the concentration of EAB and EPQ complexes is essentially zero (A and B are the substrates and P and Q the products and EAB is enzyme–substrate complex and EPQ is the enzyme product complex) (Segel 1975; Zhang et al. 2014). Sequential kinetics can be distinguished from ping-pong kinetic mechanisms by the formation and release of one product before the binding of the second substrate.

In random mechanism, there is no obligatory binding sequence and this makes the reaction mechanism much more complex. Therefore, we may predict/explain that ordered bi–bi evolve into random bi–bi catalytic mechanisms (Segel 1975). It has been suggested that promiscuous activities are common because the evolution of a perfectly specific active site is both difficult and unnecessary (Copley 2015). The non-sequential mechanism, also known as the ping-pong mechanism, does not require both substrates to bind before releasing the first product. The name refers to the way in which the enzyme bounces back and forth from an intermediate state to its standard state (Segel 1975). For example, in the aminoacylation of tRNAIle, there are four different orders of substrate addition and product release that take place via sequential ordered ter–ter, rapid equilibrium sequential random ter–ter, random bi–uni uni–bi ping-pong and bi–bi uni–uni ping-pong, with a rapid equilibrium segment, mechanisms. tRNAVal is aminoacylated in rapid equilibrium random ter–ter order via a bi–bi uni–uni ping-pong mechanism with a rapid equilibrium segment and via two bi–uni uni–bi ping-pong mechanisms. It is assumed that assay conditions can be regarded as a stepwise approximation of physiological conditions and that considerable changes in error rates, up to one order of magnitude, may be possible in vivo (Freist and Sternbach 1984). Numerous steady-state kinetic studies have examined the complex catalytic reaction mechanism of multifunctional enzymes, such as pyruvate carboxylase. This enzyme catalyses reactions through a non-classical sequential bi–bi uni–uni reaction mechanism (Menefee and Zeczycki 2014). However, in experiments of another multifunctional enzyme, enzyme fatty acyl-AMP ligase FadD33, the researchers clearly demonstrated that catalysis proceeded via a bi uni–uni bi ping-pong kinetic mechanism (Vergnolle et al. 2013). N10-formyltetrahydrofolate synthetase is a folate enzyme that catalyses the formylation of tetrahydrofolate in an ATP-dependent manner, specifically, via a random bi uni–uni bi ping-pong ter–ter mechanism (Celeste et al. 2012). Malonyl-CoA synthetase catalyses the formation of malonyl-CoA directly from malonate and CoA, with hydrolysis of ATP into AMP and pyrophosphate (PPi). The catalytic mechanism of malonyl-CoA synthetase was investigated in steady-state kinetics and initial-velocity and product inhibition studies with AMP and PPi. The results strongly pointed to an ordered bi uni–uni bi ping-pong ter–ter system as the most probable steady-state kinetic mechanism of malonyl-CoA synthetase (Kim and Kang 1994).

Enzyme kinetic mechanisms are specific to their substrates because of their functional specificity. Determining enzyme functions is essential for a thorough understanding of cellular processes. The functional specificity of an enzyme can change dramatically following the mutation of a small number of residues. Information about these critical residues can potentially help discriminate enzyme functions (Nagao et al. 2014). In a previous study, researchers added glycerol to their activity assay buffer, and this molecule ‘glycerol’ caused a decrease in both *K*_m_ and *K*_i_ values with respect to the enzyme’s substrate. They attributed this finding to glycerol causing a conformational change in the enzyme, resulting in tighter binding of the enzyme’s substrate and its product (Kulaksiz-Erkmen et al. 2012).

Multienzyme complexes and multifunctional proteins may confer a kinetic advantage by channelling reaction intermediates between consecutive enzymes and reducing the transient time for the establishment of steady states (Easterby 1989). Therefore, various enzymes with different catalytic functions may come together and make big complex machines or complex enzymatic reaction fabrics. One such enzyme is fungal fatty acid synthase, which has played a key role in the evolution of complex multi-enzymes. It has 48 functional domains, which are embedded in a matrix of scaffolding elements (Bukhari et al. 2014). Mechanism pathways for multi-substrate multi-product enzyme-catalysed reactions can become very complex and lead to kinetic models comprising several terms (Bornadel et al. 2013) or quite simple terms, such as random, sequential binding mechanisms (Burke et al. 2013). The most important thing is more than one enzyme come together to improve the productivity and reduce the cost of various processes. The most important point to remember is that more than one enzyme is required to produce any product.

Reaction mechanisms are diverse; substrate specificity is achieved by a diversity of not only substrate recognition, but also hydrolysis mechanisms (Arimori et al. 2011). However, it is difficult to predict which bi–bi substrate enzyme kinetic mechanisms emerged first. From an evolutionary perspective, the random mechanism may be much more evolved than the ordered bi–bi mechanisms. In the ordered mechanism, the binding of the first substrate to the enzyme’s active site causes a conformational change, which is required for binding the second substrate. Alternatively, the second substrate binds directly to the first substrate. If the active site of the enzyme contains various catalytic functional groups, then the substrate selectivity of this enzyme will decrease, enabling it to interact easily with various substrates, such as GST enzymes. Cytochrome p450 and GST enzymes have broad substrate specificity. They are responsible for the metabolism of non-physiological substances, such as xenobiotics. Cytochrome P450 enzymes catalyse the metabolism of a wide variety of naturally occurring and foreign compounds, via a ping-pong bi–bi mechanism. GST enzymes from humans and other sources display a random mechanism in which the combination of the enzyme with one substrate does not influence its affinity for the other (Hollenberg 1992; Breton et al. 2000; Caccuri et al. 2001; Bowman et al. 2007; Wang et al. 2011; Kolawole et al. 2011). Enzymes with promiscuous activities are also likely to have a long evolutionary history (Copley 2015).

## Conclusion

The number of substrates and the type of enzymatic reaction mechanism provide clues about the evolutionary order of an enzymatic reaction. Enzymes use various kinds of substrate analogues or slightly different substrates, which correspond to the variability of the kinetic mechanisms used to generate a product.

The possible order of bi–bi kinetic mechanisms from evolved to unevolved is random, branched, ordered and ping-pong. Promiscuity has significant roles and functions in the evolutionary steps. Promiscuous functions offer a wide range of opportunities to enzymes. A more promiscuous enzyme kinetic mechanism, such as the ability of substrates to bind to the active site via a random bi–bi mechanism, signifies that the enzyme is more evolved than, for example, an enzyme with an ordered kinetic mechanism.

Every single molecule has an evolutionary purpose and numerous cellular roles responsible for the functional operation of a given organism. However, shifting environmental conditions, ageing, exposure to mutagenic toxins, accumulation of reactive oxygen species and their insufficient neutralisation in cell can modify gene expression, which, in turn, can alter enzymes and their kinetic behaviours.

We know that life depends on the combined power of enzymes with toxic or nontoxic compounds and the synthesising of products according to cellular needs.

Evolutionary processes may give rise to diversity in enzyme kinetic mechanisms. Enzymes are not passive targets of environment changes. To understand the evolutionary steps of enzyme kinetic mechanisms, kinetic mechanisms need to be explained, beginning with those of prokaryotic organisms and culminating with those of eukaryotes.
